# Double-Labeling Method for Visualization and Quantification of Membrane-Associated Proteins in *Lactococcus lactis*

**DOI:** 10.3390/ijms241310586

**Published:** 2023-06-24

**Authors:** Mai Ngoc Hoang, Clemens Peterbauer

**Affiliations:** 1 Institute of Immunology, Department of Human Medicine, Carl von Ossietzky University of Oldenburg, 26129 Oldenburg, Germany; mai.hoang@uni-oldenburg.de; 2 Institute of Food Technology, Department of Food Science and Technology, University of Natural Resources and Life Sciences, 1190 Vienna, Austria

**Keywords:** *L. lactis*, biarsenical labeling, nickel NTA, visualization, quantification, membrane proteins, post-translational pathway

## Abstract

*Lactococcus lactis* displaying recombinant proteins on its surface can be used as a potential drug delivery vector in prophylactic medication and therapeutic treatments for many diseases. These applications enable live-cell mucosal and oral administration, providing painless, needle-free solutions and triggering robust immune response at the site of pathogen entry. Immunization requires quantitative control of antigens and, ideally, a complete understanding of the bacterial processing mechanism applied to the target proteins. In this study, we propose a double-labeling method based on a conjugated dye specific for a recombinantly introduced polyhistidine tag (to visualize surface-exposed proteins) and a membrane-permeable dye specific for a tetra-cysteine tag (to visualize cytoplasmic proteins), combined with a method to block the labeling of surface-exposed tetra-cysteine tags, to clearly obtain location-specific signals of the two dyes. This allows simultaneous detection and quantification of targeted proteins on the cell surface and in the cytoplasm. Using this method, we were able to detect full-length peptide chains for the model proteins HtrA and BmpA in *L. lactis*, which are associated with the cell membrane by two different attachment modes, and thus confirm that membrane-associated proteins in *L. lactis* are secreted using the Sec-dependent post-translational pathway. We were able to quantitatively follow cytoplasmic protein production and accumulation and subsequent export and surface attachment, which provides a convenient tool for monitoring these processes for cell surface display applications.

## 1. Introduction

*Lactococcus lactis*, a naturally safe microorganism that is consumed with many food products, has emerged as a potential drug delivery vehicle. Live-cell *L. lactis* bacteria displaying recombinant tetanus toxin fragment C (TTFC) efficiently triggered systemic immune responses against tetanus upon oral administration in mice [1]. A biosynthetic antigen carrying pneumococcal type 3 capsular polysaccharide (CPS) was expressed on the surface of *L. lactis* and was capable of immunizing mice as effectively as the application of an equivalent amount of purified antigen [2]. The expression of pneumococcal antigens on *L. lactis* is even more prevalent than those on *E. coli* due to higher stability achieved after genetic manipulations [2,3]. An engineered surface displayed rotavirus outer shell protein VP7 in *L. lactis* generated neutralizing antibodies against rotavirus in mice, even though its secreted form appeared to be a stronger immunogen [4]. *L. lactis* showed potential for live-cell HIV vaccine delivery after both mucosal and cell-mediated immune responses were developed following oral vaccination of *L. lactis* displaying the HIV envelope protein on the cell surface [5].

*L. lactis* is a small, non-sporulating, immotile, and cocci-forming bacterium with a regular diameter in the range of 0.5–1.5 µm. The localization of molecules such as recombinant proteins is greatly challenged by the overall tiny size. Robust and precise methods for visualization and quantification of recombinant proteins are required for tracking the localization of targeted proteins and evaluating the efficiency of the expression approach. The most common approach for such purposes is currently fractionating the producing cells, which is laborious and time-consuming and often not sufficiently accurate [6,7]. A series of conventional laboratory methods are needed, including biomass homogenization, mass separation by sodium dodecyl sulfate polyacrylamide gel electrophoresis (SDS-PAGE), immunoblotting, and size exclusion chromatography (SEC). Membrane-integrated and membrane-bound proteins are known for their hydrophobic nature, which poses problems for separation and quantification by Western blot and requires optimization [8]. An alternative approach is to fuse the proteins of interest with one of the numerous fluorescent reporter proteins, such as green fluorescent protein (GFP) and its derivatives, such as cyan, yellow, and red fluorescent protein (CFP, YFP, and RFP, respectively). However, the 27–29 kDa size of these fluorescent markers remains a challenge for their use as fusion partners in bacterial platforms. In many cases, the export of GFP-fused proteins is hindered [9], or the formation of inclusion bodies and incomplete proteins is observed [10,11].

In this study, we propose a double labeling approach to follow the expression and secretion of membrane-anchored proteins in *L. lactis*. Both homologous proteins in this study naturally exist in *L. lactis* as membrane-attached proteins, but their association with the bacterial surface is formed via different anchoring motifs [12]. BmpA is classified to be a lipoprotein which is covalently linked to the phospholipid bilayer of the cell membrane [12], while HtrA is reported to be a transmembrane protein containing a single trans-membrane segment (TMS) of 20 amino acid residues in length [13,14]. In Gram-positive bacteria such as *L. lactis*, which do not possess an outer membrane, only a thin space on the outside of the cell membrane is considered to be a “pseudo-periplasm”, and this is where HtrA is predicted to reside [14]. Our methodology used a hexahistidine label for surface-exposed target proteins, which can be bound by a Ni(II)nitrilotriacetic acid (Ni-NTA)—Atto 488 conjugate, and a tetracysteine label, which can be bound by membrane-permeable resorufin arsenical hairpin binder (ReAsH), to target intracellular proteins in living cells [15]. The former label enables us to confirm the destination of target proteins, while the latter allows us to detect target proteins in the cytoplasm prior to translocation and to follow unwanted intracellular accumulation. This allows differentiation between co-translational translocation (where no full-length peptides should be present in the cytoplasm) and post-translational translocation, where full-length peptides are transported through the translocon pores. Using this method, we could simultaneously quantify protein accumulation in the cytoplasm and surface display in *L. lactis*, which allows applications for the comparison of protein production and secretion/surface display between different cell lines.

## 2. Results

### 2.1. Overproduction and Overexpression of Recombinant Homologous Proteins

**Figure 1:**
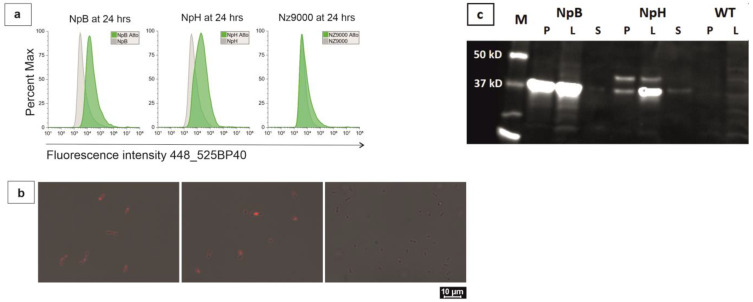
Overexpression and expression of recombinant proteins. (**a**) Surface expression of polyhistidine-tagged proteins, displayed as comparative histograms of unstained (grey) and stained (green) populations; (**b**) intracellular expression of tetracysteine tagged proteins, displayed as scaled viewer between brightfield and red fluorescence channels, from left to right: NpB, NpH, and NZ9000; (**c**) immunostaining of overexpressed BmpA and HtrA on Western Blot. P = precipitated pellet after sonication, L = cell lysate after sonication, and S = supernatant of spent media.

Overexpressed HtrA and BmpA were detected in the disrupted biomass and visualized with immunodetection (Figure 1c). For BmpA, 38.46 kDa bands were observed in the fractions derived from lysed cells but not in the concentrated supernatant of spent media. For HtrA, besides the 45.25 kDa/36 kDa double bands in lysate and cell-pellet factions, a 36 kDa band also appeared in the supernatant (likely corresponding to the protein without its transmembrane anchor). This observation verified that both HtrA and BmpA were integrated correctly in-frame and were overproduced in the nisin-induced system. The apparent single bands of HtrA and BmpA confirmed that the integrity in the cytoplasm and on the cell membrane of both proteins was preserved.

The majority of stained-induced cell populations overproducing BmpA (NpB for short) and HtrA (NpH for short) exhibited higher surface fluorescence compared to the stain-free induced controls (Figure 1), which was not seen in plasmid-free and empty-vector NZ9000 controls. Intracellular fluorescence was observed in ReAsH-stained induced NpB and NpH cells but not in both stained uninduced cells or unstained induced cells. These fluorescence signals indicated that the anchoring and exposure of recombinant proteins were completely implemented on the surface of the cells.

**Figure 2:**
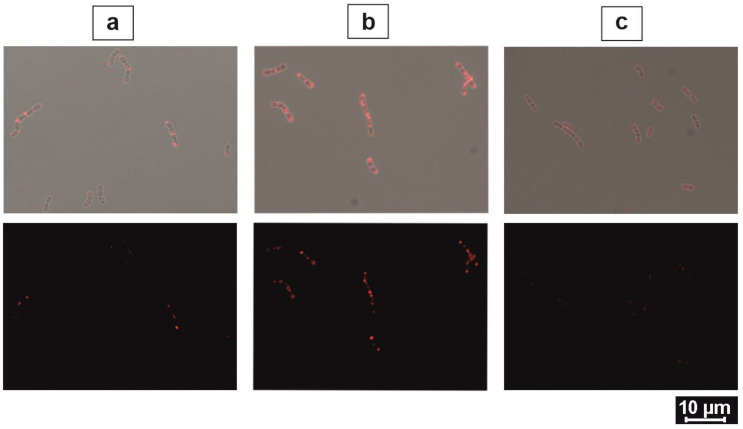
Intracellular fluorescence of NpB at different time points. Top panel: bright field channel; bottom panel: fluorescent channel. (**a**) At 1 h post-induction: fluorescence signals occurred slightly and only at the cell tips. (**b**) At 3 h post-induction: stronger fluorescence accumulation at cell tips and weak red smears appeared between the intense red spots. (**c**) At 24 h post-induction: fluorescence signals diffused equally and became faded at the cell–cell conjunction.

The fluorescence exhibition of NpB and NpH cells appeared differently. Only cells carrying recombinant BmpA showed fluorescence concentrated around the cell tips in the early hours, whereas NpH cells expressed intense fluorescence in single or short-chain cells and not specifically on chain tips (Figure 1b). Particularly, BmpA was accumulated increasingly from 1 to 3 h post-induction and was eventually distributed evenly at 24 h (Figure 2). The microscopic presentation of three constitutive staining applications (Ni-NTA-Atto 488, ReAsH, and DAPI) provided rich details about the trafficking of membrane-associated proteins.

### 2.2. Trafficking of Membrane-Associated Proteins in Live Bacterial Cells

**Figure 3:**
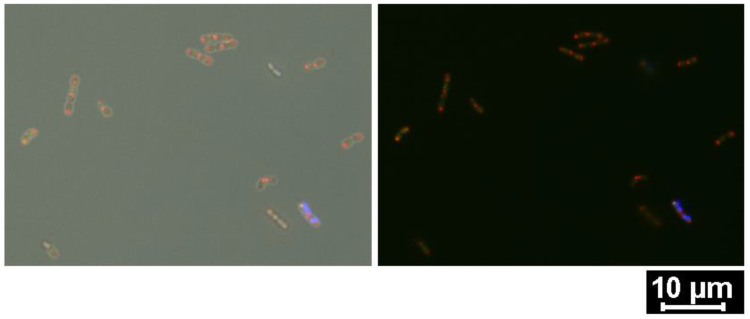
Multi-channel microscopic display of NpB at 3 h post-induction. Intracellular BmpA was accumulated at the cell tips (red), surface-anchored BmpA exhibited slight green fluorescence along the whole cell (green), and the nuclear area was condensed in the cells and seemingly separated from the cell tips (purple–blue). Overlay of three fluorescence channels with (**left** image) and without (**right** image) bright field channel.

The localization of BmpA, from premature unfolded peptides to mature proteins, could be spotted in live cells (Figure 3). Our staining strategy worked effectively in visualizing nano-sized elements at different developmental stages and performed precisely for simultaneous detection in different cell compartments.

### 2.3. Optimization of Biarsenical Technology for Membrane-Associated Proteins

**Figure 4:**
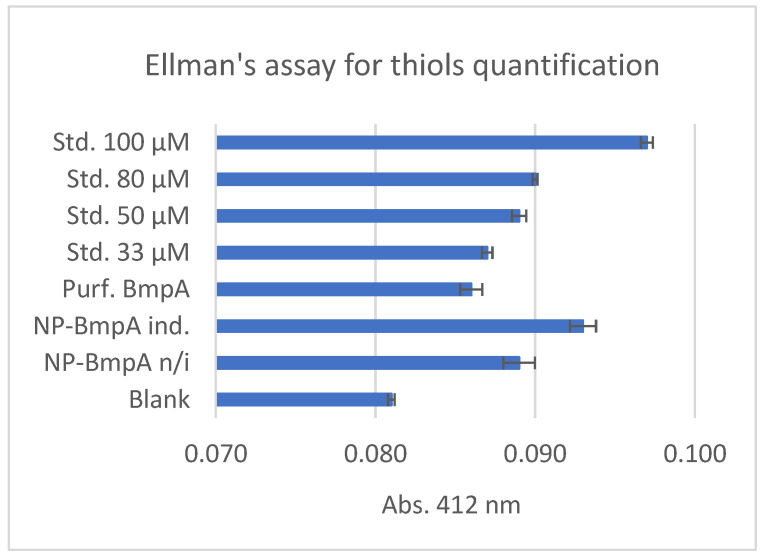
Ellman’s assay for sulfhydryl group quantification in bio-organism samples. The comparison was made among lysate of disrupted NpB cells, non-induced cells, induced cells, purified TC tagged BmpA, and acetyl cysteine standards at 33–50–80–100 μM. Standard curve and figures of protein purification can be found in Supplemental Figure S3.

Ellman’s assay showed that absorbance values of purified BmpA, non-induced biomass, and induced biomass were significantly higher than that of blank solution (Figure 4). The reaction of DTNB with induced NpB biomass resulted in higher absorbance values compared to non-induced ones. This result suggested that the induced sample contained more free thiols, and this extra amount of thiol groups caused the measurable difference in Ellman’s assay. The purified protein showed a poorer absorbance value compared to both biomass samples. This is reasonable because this sample contained only BmpA, while the other two were supposed to accommodate a great number of proteins.

In vivo examination showed that NpB cells treated with higher doses of DTNB displayed lower ReAsH-TC fluorescence. The most suppressed fluorescence was observed in NpB treated with DTNB 4 mM, which was the saturated level of DTNB in working buffer (PBS, pH 7.28) (Supplemental Figure S2). Extra washing steps did not change the fluorescence level of NpB. These results imply that the amount of DTNB introduced to TC-tagged protein carriers affected ReAsH-TC fluorescence in a dose-dependent manner.

**Figure 5:**
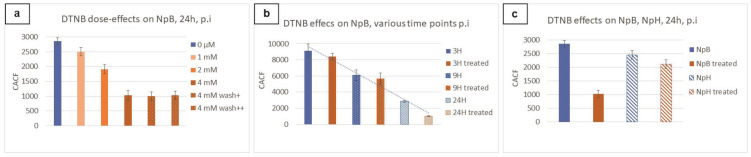
Effects of Ellman’s reagent treatment on *L. lactis* cells in vivo. (**a**) Different doses of DNTB on NpB cells 24 h post-induction. (**b**) Effects of treatment with 4 mM DTNB on NpB cells at different time points. (**c**) Effects of treatment with 4 mM DTNB on NpB and NpH at 24 h post-induction (p.i). CACF: calibrated average cell fluorescence.

**Figure 6:**
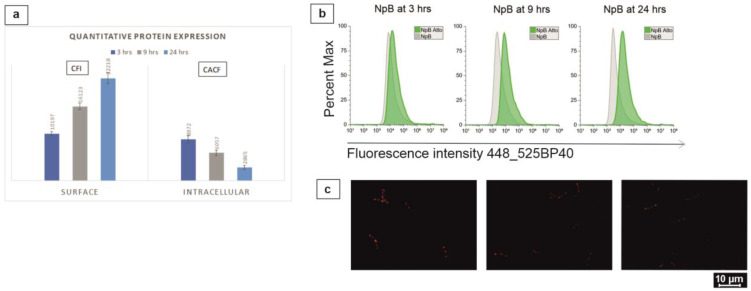
Quantitative comparison on the surface and intracellular fluorescence expression of NpB at 3-h, 9-h, and 24 h post-induction. (**a**) Quantitative comparison. (**b**) Corresponding representation of flow cytometric histograms. (**c**) Corresponding representation of microscopic observation. CFI: calibrated median fluorescence intensity. CACF: calibrated average cell fluorescence.

We compared the performance of NpB cells collected at 3, 9, and 24 h untreated or treated with 4 mM DTNB (Figure 5b). All samples treated with DTNB displayed lower fluorescence compared to their regularly stained counterparts. Remarkably, the fluorescence strength in DTNB-treated and untreated NpB at 3 h and 9 h were slightly different, but DTNB-treated NpB at 24 h showed significantly lower fluorescence. This, together with the highest surface fluorescence acquired in flow cytometry, led to the assumption that Ellman’s reagent effectively prevented the reaction between ReAsH-EDT2 and surface-anchored TC-tagged proteins by chemically blocking free thiols of TC domains. Further examination was carried out to verify this phenomenon in *L. lactis* strain carrying recombinant HtrA. The reduction in fluorescence signals of DTNB-treated NpH compared to untreated ones was detected (Figure 6c).

### 2.4. Quantification of Overexpressed Proteins

The levels of protein expression on the surface and inside the cells were quantified, and these measurements reflected corresponding visual observations on a comparative scale (Figure 6). Fluorescence exposure on the NpB surface progressed from 3 to 24 h after induction, which was shown by the increasing gap sizes between the peaks in the histograms and indicated by the quantitative fluorescence intensity (CFI value) (Figure 6a,b). In contrast, intracellular fluorescence deteriorated significantly over time. The microscopic monogram channel determined visually that NpB cells harbored the highest level of TC-tagged proteins inside the cytoplasm at 3 h post-induction, less after 9 h, and lowest at 24 h (Figure 6c).

## 3. Discussion

### 3.1. Real-Time Detection and Quantification of Protein Expression in L. lactis Cytoplasm and on Cell Surface

Flow cytometry has been proven to be an important tool for the quantification of cell-based protein expression, especially for cell surface-associated proteins [16]. The use of flow cytometry is more suitable for quantitative measurements of certain bacteria than for others due to size and colonial attachment. Theoretically, the majority of bacteria are suitable for flow cytometry screening because most of them are 0.5–2 μm in diameter, fitting in the ideal range of 0.2–150 μm for flow-cytometry-based assays [17]. For instance, *E. coli* is typically 0.75 μm [18], *Lactobacilli* are 0.7–1.5 μm [19], and *L. lactis* is 0.5–1.5 μm. Nevertheless, some bacteria might go through endospore developmental periods resulting in populations with dimensional variations. Movements by flagella, if present, may cause entanglement of cells and cause clump formation. In addition, chain-forming bacteria, such as *Streptococcus* spp., *Lactobacillus* spp., or *L. lactis*, can exist in various chain lengths, and signal intensity may become affected by chain length rather than by the signals of single cells. *L. lactis* bacteria do not show size, sporulation, or motility issues; however, they naturally appear both as single cells and as chains of cocci. Here, we observed that under light microscopy, nearly all *L. lactis* cell strains appeared as single cells or short chains of two cells, including the plasmid-free NZ9000, NpH, strains carrying heterologous protein and mutations. The appearance of four-cell chains was sporadically spotted. NpB cells notably formed longer chains of four to eight cells and were rarely seen as single cells. The object-dependent gating strategy is essentially important to eliminate incomparable minor populations and only acquire automated fluorescence intensity of the major population with comparable forward scattering value (Supplemental Figure S1).

The existence of a rather substantial peptidoglycan cell wall in *L. lactis* was shown to limit its detectable surface display in the flow cytometric platform, even though fractional immunoblotting confirmed expression [12,20]. In preliminary experiments, fluorescence shifts of His-tagged HtrA and BmpA on the surface of induced NpH and NpB, respectively, were hardly distinguishable from the controls when we applied immunolabeling techniques using mouse monoclonal immunoglobulin IgG1 to penta-histidine and polyclonal rabbit anti-mouse immunoglobulin, even though the expression was clearly confirmed in Western Blot assays. The utilization of the Ni-NTA-Atto 488 labeling factor resolved this non-correlation between Western Blot assay and flow cytometry assay. Apparent fluorescent shifts were consistently observable and repeatable in induced NpH and NpB samples (but not in uninduced samples and induced empty-vector or vector-less NZ9000). We suppose that the modest size of Ni-NTA-Atto 488 conjugate (Nickel nitrilotriacetic acid: 247.82 g/mole, Atto 488: 576.6 g/mole–PubChem Database) is beneficial for approaching and detecting membrane-associated proteins, rather than the larger conjugated antibodies. The quantity of expressed proteins is interpreted through the acquisition of Atto 488 signal by the analysis workstation, and the intensity of fluorescent signals transmitted is positively correlated to the abundance of polyhistidine-tagged surface attached proteins on individual entities which pass through the light beam. This combination of staining strategy and data analysis methods allows straightforward quantitative evaluation between samples or between different time points of each sample.

In addition to the popular usage of fluorescence microscopic methodologies in qualitative assays, pixel-based quantification of fluorescent spots has been used as a method for the demonstration of labelled protein expression levels [21,22,23]. Many of these techniques require the manipulation of bioinformatic tools and algorithms with the support of advanced microscopic systems, such as time-lapse microscopy, fluorescence recovery after photobleaching (FRET), and image-based fluorescence correlation spectroscopy (FCS) [23]. Our calibrated average cell fluorescence (CACF) value, which was adapted from the published quantitative evaluation of corrected total cell fluorescence (CTCF) [24], is reflective and manually possible. The total cell fluorescence value considers every cell within the image frame, leading to the requirement of balancing cell numbers before quantitatively comparing between different samples. This manipulation could result in bias and confusion, especially for microorganisms which are small, reproduce fast, and may be aggregated. To compare the protein expression ability of different cell lines, the average cell fluorescence used to calculate mean fluorescence intensity (MFI) is more valid because this evaluation is cell-line representative and independent of cell numbers existing in each microscopic frame. Our results showed that the MFI value comparably represented the visual evaluation of pixel-based fluorescence intensity, which correlated with the intracellular expression of targeted proteins.

Altogether, our labeling strategy and quantitative methods enable a simultaneous quantification of protein display on the cell surface and full-length peptide chains in the cytoplasm in *L. lactis*. Furthermore, the trafficking and localization of proteins during expression are visually apparent using this method.

### 3.2. Ellman’s Reagent in Biarsenical Labeling Technology

**Figure 7:**
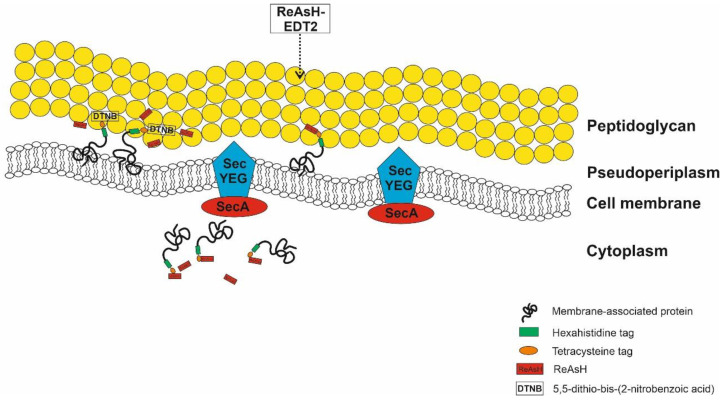
Schematic depiction of ReAsH-TC binding for membrane-anchored proteins translocated by the post-translational pathway in *L. lactis*. When a certain protein is translocated using the post-translational mode, its full-length preproteins appear in the cytoplasm and on the cell surface. The co-binding of ReAsH to surface-anchored TC-tagged proteins can be theoretically restricted by preventing contact between ReAsH with TC on the surface (using DTNB as an example in this case). The scheme is illustrated by the authors using CorelDraw.The employment of Ellman’s essay is a solution to overcome this problem. In principle, Ellman’s reagent, 5,5′-dithio-bis-(2-nitrobenzoic acid) (DTNB), is able to react with free sulfhydryl groups to release a molecule of 2-nitro-5-thiobenzoic acid (TNB) and a mixed disulfide [30]. Ellman’s assay is the classical method for the detection and quantification of sulfhydryl groups in chemical and biological samples developed by George L. Ellman [31]. DTNB absorbs weakly at 412 nm wavelength, but its daughter anion 2-nitro-5-thiobenzoate (also known as Ellman’s anion, or abbreviated as TNB^-^) has an excellent molar extinction coefficient (ε) at 412 nm of 13,600 M^−1^ cm^−1^ at pH 8.0 [31] or 14,100 M^−1^ cm^−1^ at pH 7.3 [32]. DTNB reacts stoichiometrically with free thiols existing in biomolecules to release TNB^−^, which can be precisely quantified in spectrophotometric platforms.

The advancement of biarsenical dyes in fluorescent complexes with tetracysteine (TC)-tagged proteins has been beneficial for a variety of applications on live cell screening [25,26,27], analytes, and extracted molecules [28,29]. Most prominently, the dye’s property of penetrating intact cell membranes enables highly sensitive visualization of tagged proteins in intracellular compartments of living objects as well as extracellularly secreted proteins. The situation is more complicated for membrane-associated proteins. The coexistence of TC-tagged membrane proteins on both surface and intracellular compartments depends on several factors, including the type of translocation pathway and the developmental stage of cells. If the cells use the signal recognition particle (SRP) dependent cotranslational pathway for a given protein, full-length translated peptide chains will not be present in the cytoplasm, and a C-terminally attached tag will not be detectable. However, if the targeted protein follows a post-translational pathway, fully translated proteins exist in both compartments, and their prevalence of occupation depends on the developmental stage of the cell. Their dominance inside the cells should be higher in the starting hours after induction and lower when production and translocation processes have delivered most or all the overproduced proteins to their designated location. The residence of TC-tagged proteins on the cell surface and in the cytoplasm might lead to inseparable fluorescence acquisition which comes from either compartment (Figure 7). This implies challenges for the quantitative evaluation of proteins uniquely localized in each division.

The CCXXCC tag is a small sequence but abundant in cysteine molecules and able to covalently bind to two arsenical atoms which stay correspondingly distant from each other [33]. Its modest size (<0.7 kDa) and permeability allow efficient binding to intracellular proteins in intact cells [34]. The reaction between DTNB and free thiols of surface-anchored TC-tagged proteins is expected to lead to the on-site occupation of mixed disulfide, and this newly formed compound is expected to be adequately stable to occupy the coupling clamps of the TC-tag prior to its exposure to ReAsH. DTNB is a non-permeable reagent and will not react with cytoplasmic proteins [35]. Therefore, ReAsH can bind to intracellular proteins, but undesirable co-binding to membrane-anchored proteins can be prevented. The application of Ellman’s reagent for this specific purpose was successful in both in vitro and in vivo assays and was adequately effective in occupying free sulfhydryl groups of TC-tagged proteins exposed on the surface of *L. lactis*, allowing more accurate quantification of intracellular fluorescence intensity without interferences from tagged membrane proteins.

### 3.3. Determination of Translational Pathways for Membrane Associated Proteins in L. lactis

Our fluorescence detection strategy revealed important findings about the transportation pathway that *L. lactis* used for membrane-anchored proteins. For surface expression, the majority of both stained NpB and NpH cells emitted higher fluorescence compared to their stain-free controls post-induction (Figure 1). For intracellular signals, red fluorescence was observed in NpB and NpH cells stained with ReAsH. The fluorescence acquired on the cell surface indicated that the anchoring and exposure of recombinant proteins was successful. Additionally, the appearance of labelled proteins in the cytoplasm demonstrated that full-length peptide chains were established inside the cells before being translocated to the outer side. In *E. coli*, approximately 1.000 inner-membrane proteins are directed to their locations by the co-translational pathway [36,37,38], while the post-translational pathway translocates the majority of secretory proteins of *E. coli*, approximately 400–500 proteins [39]. During co-translational translocation, translation of the mRNA by the ribosome is interrupted upon binding of the emerging signal peptide by the signal recognition particle until the ribosome is attached to the Sec translocon and resumes by “feeding” the nascent polypeptide chain directly into the translocon pore, from which it emerges on the outer face of the membrane [40]. In this scenario, the C-terminal TC-tag should be occluded inside the translocon pore upon leaving the ribosome and not be accessible and detectable by ReAsH in the cytoplasm. The detection of cytoplasmic full-length peptide chains indicates for the first time that the Sec post-translational pathway is responsible for the secretion of membrane-associated proteins in *L. lactis*.

## 4. Materials and Methods

### 4.1. Genetic Designation and Engineering

**Figure 8:**
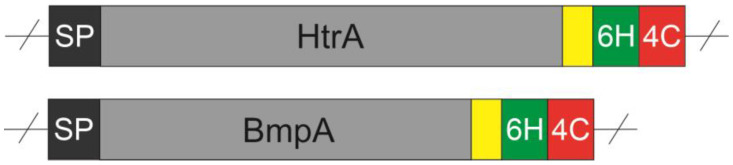
Structural design of engineered gene constructs. 6H: hexa-histidine tag, 4C: tetra-cysteine tag, blue patch: GSG linker, SP: signal peptide.

Housekeeping protease A HtrA gene (LLNZ_12505, protein ID: ADJ61386.1) and basic membrane protein A BmpA gene (LLNZ_05500, protein ID: ADJ60067.1) were isolated from *L. lactis* strain NZ9000 using polymerase chain reaction (PCR), then flanked with *Sca*I/*Sac*I and *Sca*I/*Hind*II restriction sites, respectively. A short linker (GSG), a hexahistidine tag (HHHHH), and a tetracysteine tag (CCPGCC) were inserted downstream of the gene and upstream of the C-terminal restriction site (Figure 8). The nisin-inducible gene expression (NICE^®^) system with plasmid pNZ8150 (MoBiTec GmbH, Göttingen, Germany) was used to control the expression of proteins of interest. Engineered genes and plasmid pNZ8150 were digested with corresponding endonucleases, then ligated using T4 ligase to result in pNZ8150:HtrA and pNZ8150:BmpA vectors following protocols provided by the manufacturer (New England Biolabs, Ipswich, MA, USA).

### 4.2. Cell Culture and Plasmid Transformation

*L. lactis* NZ9000 cells (MoBiTec GmbH) were cultured in M17 media supplemented with 0.5% glucose (GM17) at 30 °C without agitation. Recombinant-proficient (recA+) *E. coli* strains were used as intermediate vector recipients and grown in lysogeny broth (LB) media in 200 rpm shaking test tubes at 37 °C. The cells bearing plasmid pNZ8150 were grown in media supplemented with 10 μg/mL chloramphenicol. Ligated vectors were transformed into chemically competent *E. coli* MC1061 or electroporation-competent *E. coli* JM101 using MicroPulser Electroporator (Bio-Rad, Hercules, CA). After transformation, the cells were mixed with 950 μL recovery media (0.5% (*w*/*v*) yeast extract, 2% (*w*/*v*) tryptone, 10 mM NaCl, 2.5 mM KCl, 20 mM MgSO_4_, 0.5% D-Glucose (*w*/*v*), pH 7.5), incubated at 37 °C for 1–2 h before being plated on LB media solid plates containing 1.5% m/v agar and 10 μg/mL chloramphenicol, and incubated at 37 °C overnight. Electrocompetent *L. lactis* NZ9000 was prepared as originally described [41]. Only pure plasmids with verified sequences were used for transformation into *L. lactis*.

### 4.3. Plasmid Selection, Isolation, and Sequencing

Colony PCR was conducted to screen viable *E. coli* transformant colonies for the presence of engineered genes using OneTaq^®^ Quick-Load^®^ 2X Master Mix following the manufacturer’s protocol (New England Biolabs, Ipswich, MA, USA). The colonies exhibiting bands at 1316 base pairs (HtrA) and 1134 base pairs (BmpA) on agarose gels were selected and inoculated in 5 mL LB media supplemented with 10 μg/mL chloramphenicol. Plasmids were isolated from 16 h cultures using Monarch^®^ Plasmid Miniprep Kit (New England Biolabs, Ipswich, MA, USA), quantified using Nanodrop 2000c (ThermoFisher Scientific, Waltham, MA, USA), and subjected to Sanger sequencing (Microsynth, Balgach, Switzerland).

### 4.4. Cell Induction for Protein Overexpression

Bacterial cells were recovered from glycerol stock and grown overnight (16 h) at 30 °C without agitation. The cells were inoculated into fresh GM17 (Cm +/−) at a ratio of 1:20 and grown until OD_600_ reached 0.5–0.6. Cell induction was initiated by adding 25 ng/mL nisin A from *L. lactis* (Sigma-Aldrich, St. Louis, MO, USA). Several negative controls (uninduced samples, uninduced NZ9000, and induced NZ9000) were included. Cells were harvested at 3 h, 9 h, and 24 h after induction and stored at 4 °C for further experiments.

### 4.5. Cell Disruption and Protein Measurement

*L. lactis* strains carrying HtrA (NpH) and BmpA (NpB) were induced and harvested for analytical assays. For each culture, a volume of 10 mL was centrifuged at 4000 rpm, 4 °C for 15 min. An aliquot of spent media was kept for protein measurement. The precipitated biomass was dissolved in 500 μL of ice-cold PBS buffer pH 7.5 and disrupted by ultrasonic homogenization on ice (Bandelin Sonopuls HD 60, Bandelin electronic GmbH & Co. KG, Berlin, Germany). The lysate was separated and stored on ice, while the cell pellet was washed three times with PBS and dissolved in 500 μL ice-cold PBS. The supernatant, lysate, and pellet fractions were measured in Bradford assays for protein quantification (Lambda 35 UV/Vis spectrometer).

### 4.6. Protein Analysis and Purification

The presence of targeted proteins was identified by mass separation and immunoblotting techniques. Each fraction of every sample was separated in a vertical electrophoresis cell system (Bio-Rad) and observed for visual bands using ChemiDoc Imaging System XRS+ (Bio-Rad). A protein marker (Precision Plus Protein™ WesternC™ blotting Dual Color standards, Bio-Rad) was included. Protein bands from SDS-PAGE were transferred onto a nitrocellulose blotting membrane (Trans-Blot^®^ Turbo™ transfer pack, 0.2 μm nitrocellulose, Bio-Rad) using a Trans-Blot Turbo Transfer system (Bio-Rad), incubated overnight with BSA 2.5% at 4 °C, subsequently incubated overnight with mouse monoclonal penta-his antibody (QIAGEN), and finally exposed to polyclonal rabbit anti-mouse antibody HRP (Dako) and precision protein streptactin-HRP (Bio-Rad). Detected proteins were visualized using ChemiDoc Imaging System XRS+ (Bio-Rad).

His-tagged recombinant BmpA protein was purified using immobilized metal affinity chromatography (IMAC). The biomass of 250 mL cell culture of 3 h induced NpB was disrupted by sonication. The cell lysate was dissolved in 30 mL Buffer A (NaCl 100 mM, Tris-HCl 50 mM, glycerol 5% (*v*/*v*), imidazole 20 mM) and loaded onto a HisTrap HP 5 mL column (Cytiva, Washington, D.C.), which was attached to an ÄKTA Go purification system (Cytiva) and monitored by UNICORN™ 7 software (Cytiva). His-tagged protein was washed off the column by 80% gradient Buffer B (NaCl 100 mM, Tris-HCl 50 mM, glycerol 5% (*v*/*v*), imidazole 500 mM). The eluted solution correlating to UV absorption was collected and checked for protein concentration before being gathered in a pooled fraction. The combined fraction containing purified protein was transferred into Amicon Ultra-15 centrifugal filter units 15,000 MWCO, and the buffer was exchanged against Storage Buffer (NaCl 100 mM, Tris-HCl 50 mM, glycerol 5% (*v*/*v*)). Protein concentration in crude extract, flow-through, collected fractions, and concentrated pooled fractions were analyzed on polyacrylamide gel. Concentrated purified TC-tagged BmpA was preserved at −80 °C in aliquots.

### 4.7. Surface Protein Staining

The concentration of 2.5 µM Ni-NTA-Atto 488 (Sigma-Aldrich) was selected as the working dose after preliminary dose testing experiments with three concentrations (1.25 µM, 2.5 µM, and 5 µM) on *L. lactis* NpB. For every sample, roughly 4 × 10^9^ cells were washed twice with 500 µL ice-cold PBS and stained with 2.5 μM Ni-NTA-Atto 488 in PBS for 1.5 h on ice. After staining, the cell pellet was washed three times with ice-cold PBS, dissolved in 100 μL ice-cold PBS, and kept on ice until screening. Unstained cells and stained cells containing empty vectors were processed in the same procedure as negative controls. All the cells were loaded into a flow cytometry system (CytoFLEX S, Beckman Coulter) for fluorescence detection with bandpass filter 488–525 nm.

### 4.8. Blocking of Surface-Anchored Tetracysteine Tags

The reaction between Ellman’s reagent (5,5’-Dithiobis (2-nitrobenzoic acid) or shorty as DTNB) and TC-tagged proteins was tested in vitro with a calibration curve using 0 μM–10 mM acetyl cysteine as the standard substrate. The absorbance values were plotted against each concentration on a linear scale, resulting in an acceptable R-Squared value (Supplemental Figure S3a). Absorbance at 420 nm of purified BmpA, non-induced biomass, and induced biomass was compared with that of blank solution (100 mM NaCl, 50 mM Tris-HCl, 5% (*v*/*v*) glycerol, pH 7.5) and a series of acetyl cysteine standards at 33–50–80–100 μM.

The effects of Ellman’s reagent on the staining of TC-tagged surface-associated proteins on living bacteria were tested by introducing a DTNB treatment prior to ReAsH staining and comparing it with cell lines not treated with DTNB. The solubility of DTNB in PBS buffer at optimal neutral pH is saturated at 4 mM; thus, a set of DTNB concentrations from 0–4 mM was introduced separately on NpB at 24 h post-induction (maximum surface display of BmpA according to preliminary observation). For each dose of DTNB, 4 × 10^9^ cells were washed twice with PBS and incubated with 1 mL DTNB solution for 45 min. NpB cells were also collected at 3 h and 9 h post-induction for testing the effects of DNTB treatment on cells at different time points. Non-DNTB-treated samples were kept as a negative control. Subsequently, a regular protocol for ReAsH staining was applied. DTNB treatment effects on *L. lactis* cells were also tested regarding irreversibility (by including one and two extra washing steps) and protein types (by including NpH).

### 4.9. Intracellular Protein Staining

Preliminarily, a range of four ReAsH-EDT2 doses (1.25 μM, 2.5 μM, 5 μM, and 10 μM) was tested on *L. lactis* NpB to investigate saturation uptake. A preliminary test for the necessity of BAL treatment was also performed on NpB cells. Roughly 4 × 10^9^ cells of each sample were washed twice with PBS before being treated with 500 mL 2,3-Dimercaptopropanolin (BAL) 650 μM in PBS for 30 min. The cell pellet was then washed twice with PBS and stained with 2.5 μM ReAsH-ETD2 (Cayman, Ann Arbor, MI) in PBS for 30 min. After staining, the cell pellet was washed two consecutive times with 500 μL BAL 250 μM in PBS before finally being dissolved in 100 μL PBS. All the incubations were maintained at 37 °C. Unstained cells, stained empty-vector cells, and stained wildtype were processed in the same procedure as negative controls. The samples were screened under a live-cell epi-fluorescence microscopy system (Leica DMI6000B), lamp EL6000, objective HCX PL APO 63×/1.40 oil with Leica N2.1 filter cube (BP 515–560). The cells can be screened in real-time or stored at 4 °C until screening time.

### 4.10. Data Analysis

Intracellularly localized proteins were quantitatively evaluated given fluorescence intensity outputs from CytExpert 2.4. Gating tools were utilized to sort out debris and incomparable events due to cell damage and the chain-forming feature of *L. lactis.* All quantitative data and dot plots were produced by CytExpert 2.4. Histogram displays were exported using Floreada (https://floreada.io/ (accessed on 15 November 2021)). The median fluorescence intensity value was calibrated following the formula: CFI = M−M_0_., where CFI = calibrated median fluorescence intensity, M = median fluorescence intensity of stained cells, and M_0_ = median fluorescence intensity of unstained cells. CFI was later used for evaluation and graphic presentation.

For intracellular fluorescence analysis, raw images of fluorescence channels were extracted from Leica Application Suite X (LAS X) software. Minimal five image acquisitions for each sample were captured at different positions on the glass slides. Visual presentations were exported from LAS X. Quantitative fluorescence analysis was based on pixel intensity on a monogram fluorescence channel and performed using Fiji. The quantification of fluorescence intensity was calculated following three steps: step 1—selection of the region of interest (ROI) on the overlay display; step 2—ROI adaption on the raw monogram display of the fluorescence channel; step 3—fluorescence intensity measurement (Supplemental Figure S1). In step 1, the ROI selection can be performed using complete manual handling using freehand tools on the overlay images or filtered semi-automatically using “auto threshold” on the monogram channel if suitable. All ROIs were selected individually to eliminate irrelevant selections using ROI Manager. Cell-free areas were randomly chosen to calculate background values. In step 2, all selected ROIs and backgrounds were consistently applied to the raw monogram images of the fluorescence channel. Fluorescence intensity was calculated for each ROI and eventually calibrated to a fixed average size of a cell, corresponding to 0.85 square pixels. The average size of a cell was determined by a preliminary calculation in three separate samples, in each of which the dimensions of 10 single cells were measured and averaged. Mean fluorescence intensity (MFI) was calculated following the formula: MFI = $\frac{\sum_{i=1}^{N}\left({CACF}_{i}\right)}{N}$, where CACF = calibrated average cell fluorescence and N = total number of ROI. CACF was calculated following the formula: CACF = $\frac{Iroi-(Aroi\times Mbg)}{Aroi}$ × 0.85, in which I_ROI_ = integrated density (IntDen) of ROI, A_ROI_ = area of ROI, and M_BG_ = mean gray value of background areas. The data presented in graphic displays are the mean fluorescence intensity (MFI) of calibrated average cell fluorescence.

## 5. Conclusions

In summary, we developed a novel double-labeling method to effectively follow the localization and transportation of membrane-anchored proteins in *L. lactis*. To overcome the limitations of the modest bacterial diameter and synthetic capacity, we employed two molecular tags, each of which are only six amino acids in length but capable of labeling proteins of interest simultaneously in the cytoplasm and on the cell surface. The combination of polyhistidine and Ni-NTA conjugated with a fluorescent bioreagent demonstrated a much higher efficiency and accuracy in signal acquisition compared to the use of immunochemical compounds. Pre-treatment with an oxidative thiol reagent (5,5′-dithiobis-(2-nitrobenzoic acid) assisted biarsenical labeling technique to track and quantify the expression of proteins inside the cells. The double-labeling method, together with fluorescence microscopy and flow cytometry, allows monitoring of the trafficking of proteins in small microorganisms (0.5 μm in size) with precision and the possibility for quantification.

## Data Availability

Materials presented here are available from the corresponding author on request.

## Supplementary Materials

The following supporting information can be downloaded at: https://www.mdpi.com/article/10.3390/ijms241310586/s1. Figure S1: Process of image handling for microscopic fluorescence quantification; Figure S2: Visually observed effects in *L. lactis* NpB at 3-hour, 9-hour and 24-hour post induction with or without pre-treatment with Ellman’s reagent; Figure S3: Supplemental data for Ellman’s assay for sulfhydryl group quantification in bio-organism samples; Figure S4, BmpA purification graph; Figure S5, AlphaFold predicted protein structure of BmpA; Supplemental Table S1, list of strains and plasmids; Supplemental Table S2, list of primers.
