# Supplementary material for: Double-Labeling Method for Visualization and Quantification of Membrane-Associated Proteins in Lactococcus lactis
Source: Int J Mol Sci. 2023 Jun 24;24(13):10586. doi: 10.3390/ijms241310586 (PMC10341821; doi:10.3390/ijms241310586)
Supplement: Supplementary file 1 [file ijms-24-10586-s001.zip › ijms-2417594-supplementary.pdf]

Supplemental Data

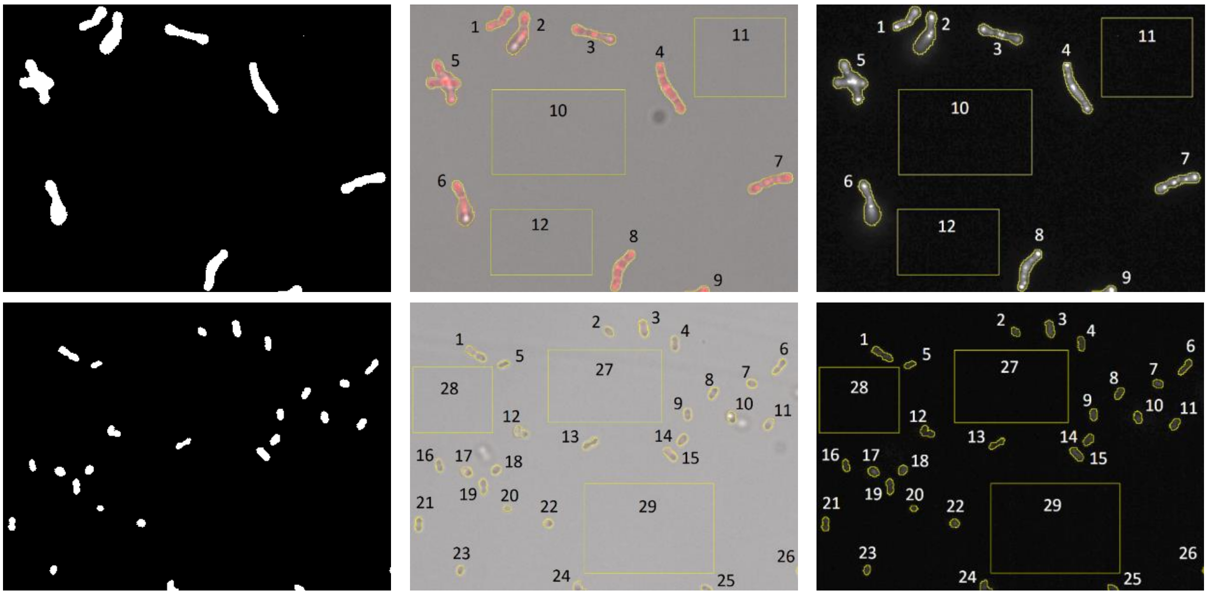

Supplemental figure S1. Process of image handling for microscopic fluorescence quantification. Upper row: NpB sample. Left: auto-threshold Li mode, middle: auto-selection of ROI from threshold and random selection of background, right: intensity measurement on raw monogram FLUO channel. Lower row: NpBE1 sample. Left: auto-threshold Max Entropy mode, middle: auto-selection of ROI from threshold and random selection of background, right: intensity statistics measurement on raw monogram FLUO channel

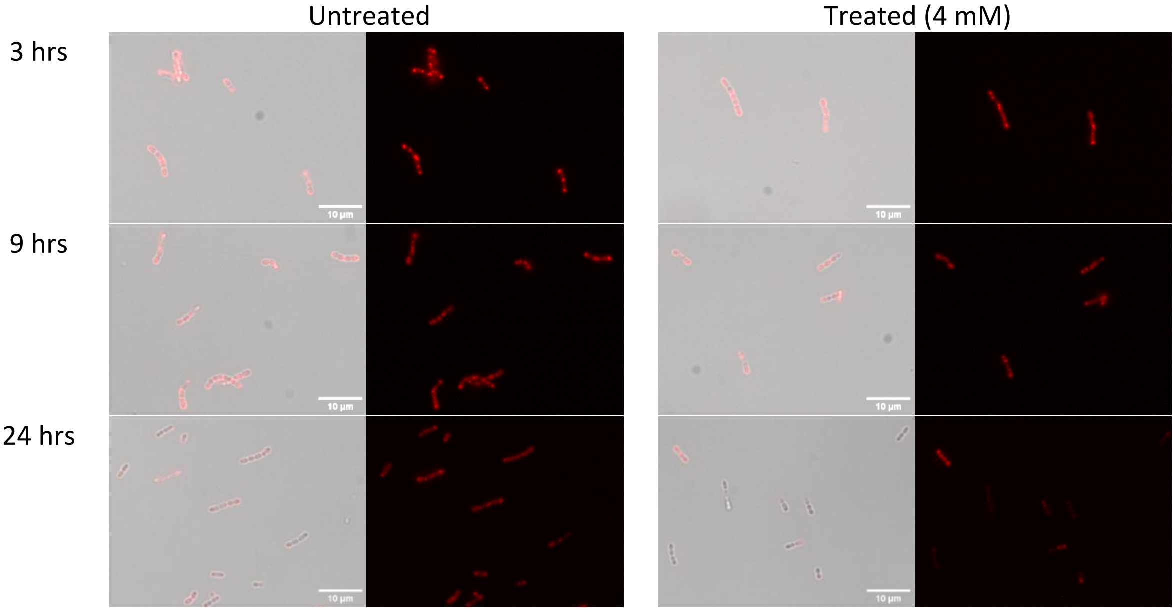

Supplemental figure S2. Visually observed effects in *L. lactis* NpB at 3-hour, 9-hour and 24-hour post induction with or without pre-treatment with Ellman's reagent (4 mM)

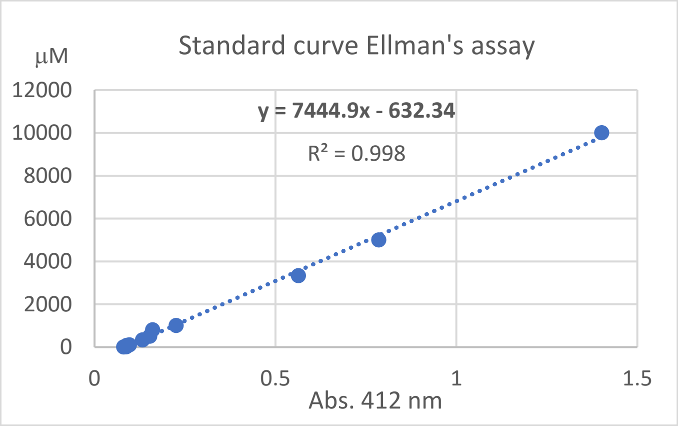

a

b

Fractions	Protein concentration (mg/ml)
Lysate	0.53
Pellet	1.42
F4	0.01
F5	0.11
F6	0.01
Flow through	0.37
Concentrated pooled F4-F6	0.28

c

50 kD
37 kD
20 kD

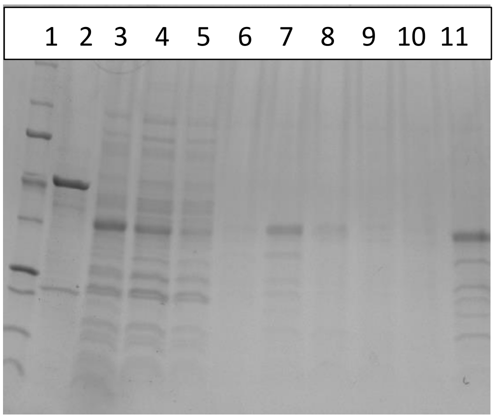

Supplemental figure S3. Supplemental data for Ellman's assay for sulfhydryl group quantification in bio-organism samples. **(a)** Standard curve of serial acetyl cysteine concentrations; **(b)** Protein concentration after IMAC purification; **(c)** Stain-free polyacrylamide gel after IMAC purification of BmpA from lysed NpB cells at 3-hour post induction, lane 2: positive control, lane 3 – 4: pellet – lysate, lane 5: flow through, lane 6 – 10: collected fractions, lane 11: buffer-exchanged concentrated pooled fraction.

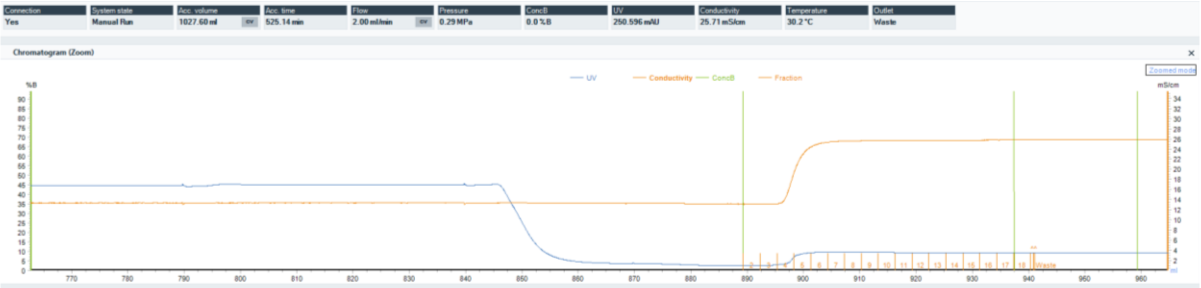

Supplemental figure S4. Graph of BmpA purification using Äkta Go and Unicorn software. A peak of protein was detected from fraction 4 and collected until fraction 8.

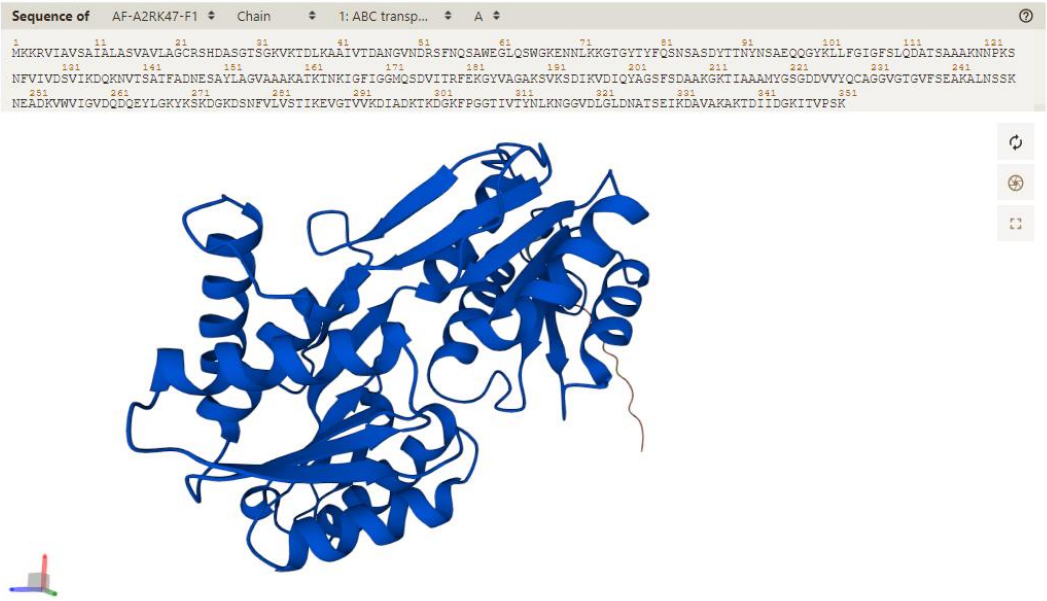

Supplemental figure S5. AlphaFold predicted protein structure of BmpA (available online at https://alphafold.ebi.ac.uk/entry/A2RK47 and Uniprot, reference number: A2RK47 - BMPA_LACLM). For predicted native localization of BmpA on *L. lactis*, refer to Berlec et. al. 2011

Supplemental table S1. List of *Lactococcus lactis* strains and plasmids

Strains	Original Strains	Plasmids	Protein of interest
NpH	NZ9000	pNZ8150:HtrA	Recombinant HtrA with 6-His & 4-Cys tags
NpB	NZ9000	pNZ8150:BmpA	Recombinant BmpA with 6-His & 4-Cys tags

Supplemental table S2. List of primers

Name	Sequence	Length	Purpose
Fw_HtrA_8150	ATTATAAGGAGGCACTCAGTACTAT GGCAAAAGCTAATATAGG	43	Amplify HtrA gene, incorporated with labelling tags.
Rv1_HtrA_8150	GGTGACCACTACCATTAGAAGAAGA TGAACTATTTGTTTCT	41	Amplify HtrA gene, incorporated with labelling tags.
Rv2_HtrA_8150	CAACAATGGTGATGGTGATGGTGAC CACTACC	32	Amplify HtrA and BmpA gene, incorporated with labelling tags.
Rv3_HtrA_8150	GCTTTTAGCAACAAGGACCGCAACA ATGGTGAT	33	Amplify HtrA and BmpA gene, incorporated with labelling tags.
Rv4_HtrA_8150	CTAATTTTGGTTCAAAGAAAGCTTTT AGCAACAAGGAC	38	Amplify HtrA gene, incorporated with labelling tags.
Fw_BmpA_8150	GAGGCACTCAGTACTATGAAAAAAC GCGTAATCG	34	Amplify BmpA gene, incorporated with labelling tags.
R1_BmpA_8150	GATGGTGACCACTACCTTTTGAAGG AACAG	30	Amplify BmpA gene, incorporated with labelling tags.
R4_BmpA_8150	AGCTTGAGCTCGCTTTTAGCAACAAG G	27	Amplify BmpA gene, incorporated with labelling tags.

Fw_8150_MSC	CTTAATTCTATCTTGAGAAAGTATTGG	27	sequencing
Rv_8150_MSC	GTAATTGCTTTATCAACTGCTGC	23	sequencing
Fw_BmpA_seq	CAGGTGTCTTCAGTGAAGC	19	sequencing
Rv_BmpA_seq	GGTCTTTAATGACAGAGTC	19	sequencing
Fw_HtrA_seq	AAGATGGTTCTACCTCTGTGG	21	sequencing
Rv_HtrA_seq	AACGGCAAGGTCAGTGTATTC	21	sequencing

Reference

Berlec A, Zadravec P, Jevnikar Z, Štrukelj B. Identification of candidate carrier proteins for surface display on Lactococcus lactis by theoretical and experimental analyses of the surface proteome. Applied and environmental microbiology. 2011;77(4):1292–300.
